# Bacterin Vaccination Provides Insufficient Protection Against *Streptococcus equi* Subspecies *zooepidemicus* Infection in Pigs

**DOI:** 10.3389/fvets.2022.827082

**Published:** 2022-02-28

**Authors:** Samantha J. Hau, Alexandra Buckley, Susan L. Brockmeier

**Affiliations:** National Animal Disease Center, Agricultural Research Service (ARS), United States Department of Agriculture (USDA), Ames, IA, United States

**Keywords:** *Streptococcus equi* subspecies *zooepidemicus*, swine, bacterin, vaccine, *Streptococcosis*

## Abstract

*Streptococcus equi* subspecies *zooepidemicus* (SEZ) is a zoonotic pathogen capable of causing severe disease in many mammalian species. Historically, SEZ has not been a common cause of disease in pigs in North America; however, in 2019, SEZ caused mortality events leading to severe illness and 30–50% mortality in exposed animal groups. Because of the rapid progression of disease, it is important to investigate intervention strategies to prevent disease development. In this study, pigs were divided into four groups: (1) vaccinated with an inactivated SEZ vaccine generated from a highly mucoid 2019 mortality event isolate; (2) vaccinated with an inactivated SEZ vaccine generated from a genetically similar, non-mucoid isolate from a guinea pig; (3) and (4) sham vaccinated. Following boost vaccination, groups 1–3 were challenged with a 2019 mortality event isolate and group 4 were non-challenged controls. Antibody titers were higher for SEZ vaccinated animals than sham vaccinated animals; however, no anamnestic response was observed, and titers were lower than typically seen following the use of inactivated vaccines. Vaccination did not provide protection from disease development or mortality following challenge, which could be associated with the comparatively low antibody titers generated by vaccination. Surviving pigs also remained colonized and transmitted SEZ to naïve contact pigs 3 weeks following challenge, indicating that healthy animals can act as a source of SEZ exposure. Future investigation should evaluate different vaccine formulations, such as increased antigen load or an alternative adjuvant, that could induce a more robust adaptive immune response.

## Introduction

*Streptococcus equi* subspecies *zooepidemicus* (SEZ) is a pathogen of horses and other mammals, including humans (1, 2). It has been infrequently associated with disease in pigs in North America (3); however, in 2019, SEZ was isolated from swine that died during high mortality events in the USA and Canada (4–6). Pigs developed weakness, lethargy, high fever, and mortality rates of 30–50% over 5–10 days following infection (6). Initial investigation into the pathogenesis of mortality event isolates revealed that SEZ is capable of causing disease in healthy, conventionally raised pigs and experimental exposure resulted in 100% mortality within 72 h post-challenge (7).

Genetic investigation of the isolates obtained from the 2019 high mortality events revealed isolates were genetically indistinguishable by phylogenetic analysis (5). Because of this, developing a vaccine capable of preventing SEZ-associated losses with a 2019 isolate should be widely effective against isolates impacting the North American swine industry. Currently, no vaccines are approved in North America to prevent SEZ in pigs and no work has investigated the use of autogenous vaccines against 2019 SEZ isolates.

The isolates obtained from the 2019 mortality events were highly mucoid, which is thought to contribute to the severe disease and rapid mortality seen in exposed animals (7); however, heavy encapsulation can also impact the response of the immune system to bacterin vaccines by blocking antigenic epitopes. Because of this, we utilized two SEZ bacterin vaccines, one generated from a highly mucoid swine mortality event isolate and the other generated from a heterologous, genetically similar but non-mucoid guinea pig isolate (5, 7). We evaluated the serological response to SEZ bacterin vaccination and evaluated the capacity of vaccination to prevent severe disease and mortality following SEZ challenge.

## Materials and Methods

### Bacterial Strains and Culture Conditions

Isolates of *Streptococcus equi* subspecies *zooepidemicus* previously evaluated for virulence were used in this study (7): swine isolate (19-031482-K1916623-LUNG1, SRR10584760, https://www.ncbi.nlm.nih.gov/sra/SRR10584760) and guinea pig isolate (19-035701-R130327313, SRR10512734, https://www.ncbi.nlm.nih.gov/sra/SRR10512734). Isolates were grown on blood agar plates (Becton Dickinson, Franklin Lakes, NJ) at 37°C with 5% CO_2_.

### Bacterin Vaccine Generation

Bacterial strains grown on blood agar plates were harvested into PBS. Bacteria were quantified by plating serial 10-fold dilutions. The bacterial suspension was formalin inactivated with addition of 10% buffered formalin to a final concentration of 0.25% formalin for 24 h at 4°C. Sterility was verified by plating on blood agar. Bacteria were washed and resuspended in PBS. Bacterin vaccines were composed of 1 × 10^9^ colony forming units (CFU) per 2-ml dose and adjuvanted with 20% EmulsigenD (MVP Adjuvants, Omaha, NE). Sham vaccines consisted of PBS with 20% EmulsigenD in 2 ml total volume.

### Animal Study

All animal studies were approved by the National Animal Disease Center Institutional Animal Care and Use Committee. Fifty-four, 6-week-old pigs were divided into four groups: group 1 pigs were vaccinated with a bacterin generated from the highly mucoid swine isolate (*n* = 15); group 2 pigs were vaccinated with a bacterin generated from the non-mucoid guinea pig isolate (*n* = 16); group 3 (*n* = 16) and 4 (*n* = 7) pigs were sham vaccinated with adjuvant only. Animals were vaccinated on day 0 and boost vaccinated on day 14. On day 28, pigs in groups 1–3 were challenged with 3 ml of 2.55 × 10^8^ CFU/ml SEZ swine isolate intranasally (1 ml per nostril) and orally (1 ml).

Animals were monitored post-challenge approximately every 4 h, excluding an 8-h overnight period, for signs of clinical disease (depression, lethargy, reluctance to rise, fever, neurologic signs). Body temperature was assessed by temperature probe implanted in the neck of each animal (Destron Fearing, South St. Paul, MN). When disease became severe, animals were euthanized. At necropsy, the following samples were collected for bacterial culture: nasal swab, tonsil swab, joint fluid, serosal swab, splenic swab, liver swab, cerebrospinal fluid, bronchoalveolar lavage fluid, and serum.

Animals were assessed for SEZ colonization by nasal and tonsil swab prior to challenge and on days 7 and 25 post-challenge (prior to commingling) in surviving animals.

### Commingling Post-challenge

On day 25 post-challenge, three non-challenged pigs (group 4) were commingled with two pigs surviving challenge (one group 2 animal, one group 3 animal) in a clean room to assess the potential for transmission of SEZ to naïve contacts. Pigs were monitored as described previously for 33 days.

### Antibody Titers

Serum was collected on days 0, 14, and 28 to evaluate vaccine response. Serum was also collected on day 53 from surviving animals (prior to commingling) and day 86 from surviving contact animals (4 weeks after commingling). All samples were stored at −80°C until ELISAs were performed. ELISAs were performed as previously described (7). Briefly, plates were coated overnight with heat-inactivated SEZ diluted 1:10 in carbonate–bicarbonate buffer. SEZ was probed with serial dilutions of serum samples and bound antibody was detected with horseradish peroxidase conjugated secondary antibody specific to swine immunoglobulin heavy chain (1:20,000 dilution) (SeraCare Life Sciences Inc., Milford, MA) and tetramethylbenzidine substrate (Life Technologies, Carlsbad, CA). Optical density was evaluated at 450 nm and data were evaluated in GraphPad Prism (GraphPad Software, La Jolla, CA) using a nonlinear function of the log_10_ dilution and log (agonist)-versus-response variable slope four-parameter logistic model. Endpoint titer was interpolated using two times the average reading of gnotobiotic swine serum.

### Statistical Analysis

Statistical assessments were performed in GraphPad Prism. Survival was assessed using the product limit method of Kaplan and Meier with comparisons using the log-rank test. Log_10_ antibody titers were evaluated using a one-way ANOVA to compare between groups on a given day and within a group between different days.

## Results

### Bacterin Vaccination Caused a Rise in Titer but No Anamnestic Response

**Figure 1:**
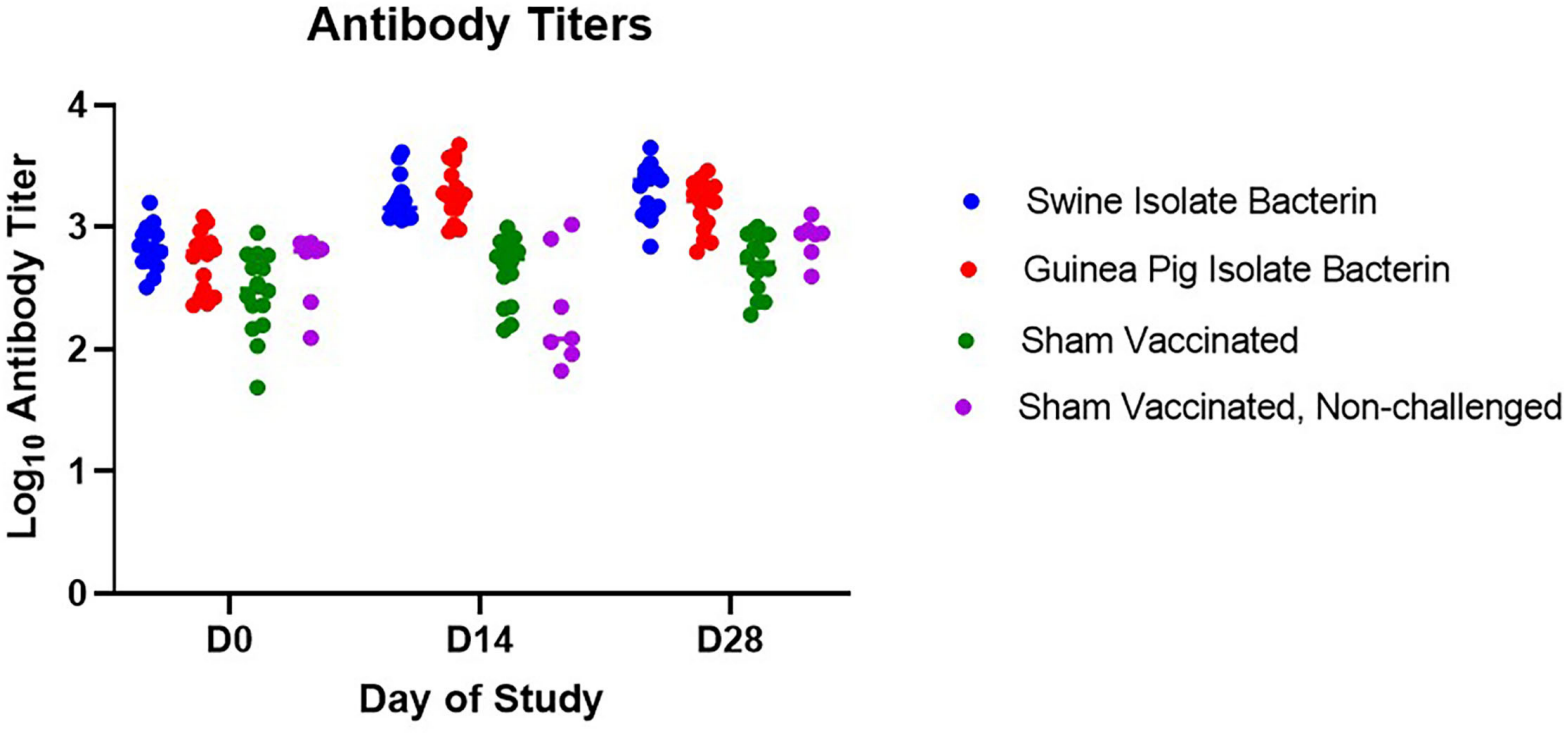
Serum antibody response to vaccination. Antibody titers for swine isolate and guinea pig isolate bacterin vaccine groups were higher than sham vaccinated animals following vaccination (days 14 and 28, *p* < 0.0001). No anamnestic response was seen following boost vaccination in either bacterin vaccine group (swine isolate, *p* = 0.6560; guinea pig isolate, *p* = 0.4123).

Serum antibody titers were assessed prior to vaccination (D0), at boost vaccination (D14), and prior to challenge (D28) (Figure 1). Following primary vaccination (D14), pigs vaccinated with both the swine isolate and guinea pig isolate bacterins had a rise in titer statistically higher than sham vaccinated controls (*p* < 0.0001). The elevation in titer was still present at challenge (D28); however, boost vaccination did not stimulate an anamnestic response and no difference in titer was noted between vaccinated pigs on D14 and D28 (swine isolate, *p* = 0.6560; guinea pig isolate, *p* = 0.4123).

### Survival Against SEZ Challenge Was Not Improved by Bacterin Vaccination

**Figure 2:**
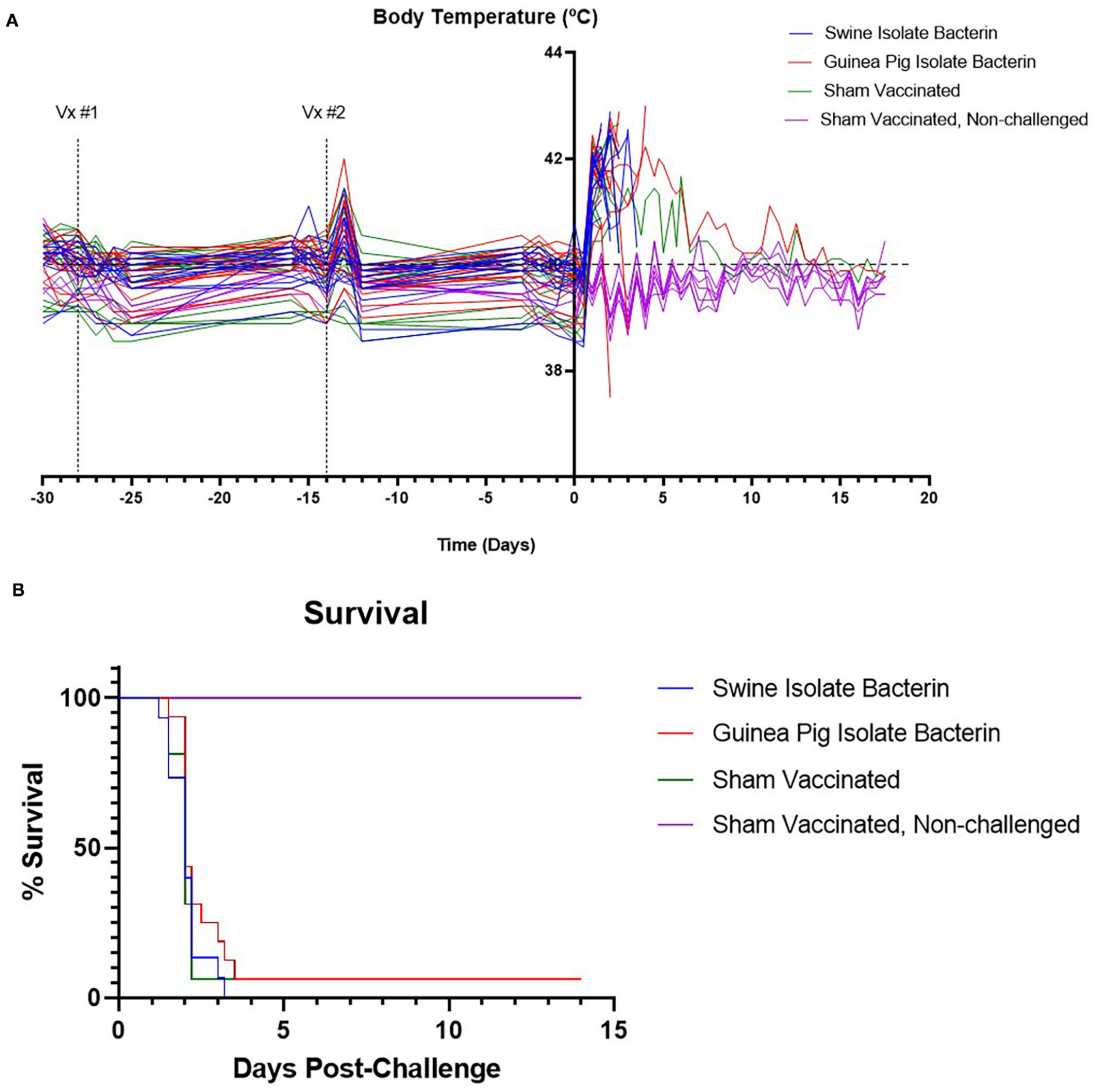
Body temperature and survival following challenge with SEZ. The temperatures of animals are plotted in **(A)**. All animals developed a fever following challenge with SEZ. The upper limit for normal pig body temperature is marked by the dashed line (40°C). Survival percent following challenge with SEZ is plotted in **(B)**. No differences in survival were observed between SEZ challenged groups regardless of vaccination status.

Animals in groups 1, 2, and 3 were challenged intranasally and orally with the SEZ swine isolate to evaluate vaccine efficacy. Pigs in all challenge groups developed clinical signs by 24 h post-challenge, including depression, anorexia, reluctance to rise, and fever (Figure 2A). By 96 h post-challenge, 15/15 pigs vaccinated with the swine isolate bacterin, 15/16 pigs vaccinated with the guinea pig isolate bacterin, and 15/16 sham vaccinated pigs were euthanized due to worsening disease (Figure 2B), revealing no effect of vaccination on clinical disease or mortality.

Surviving animals were assessed on days 7 and 25 post-challenge for nasal and tonsil colonization. No SEZ was isolated from the surviving guinea pig isolate bacterin vaccinated animal at day 7 or 25 post-challenge. The surviving sham vaccinated animal was found to have SEZ in both the nasal and tonsil sample at 7 days post-challenge, but SEZ was not detected 25 days post-challenge.

### Animals Surviving SEZ Challenge Remain an Exposure Source for Naïve Animals 25 Days Post-challenge

**Figure 3:**
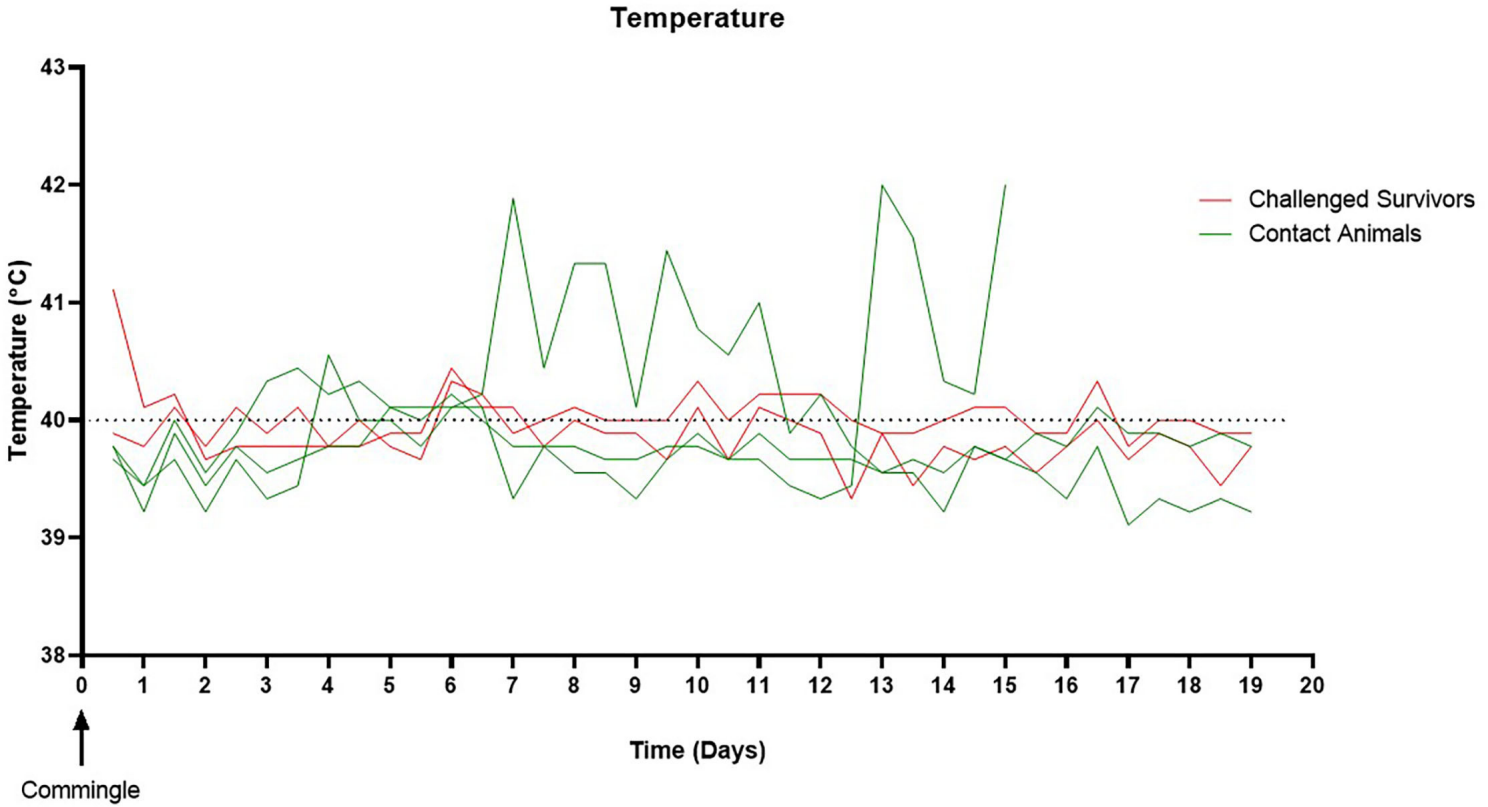
Pig temperature following commingling. All naïve contacts were febrile at one or more time points post-commingling. Two pigs were febrile for multiple readings. One pig developed a fever on day six post-commingling, which remained elevated for 4 days then returned to normal. One pig became febrile on day 12 post-commingling, developed further clinical signs, and was euthanized 15 days post-commingling. The upper limit for normal pig body temperature is marked by the dashed line (40°C).

**Figure 4:**
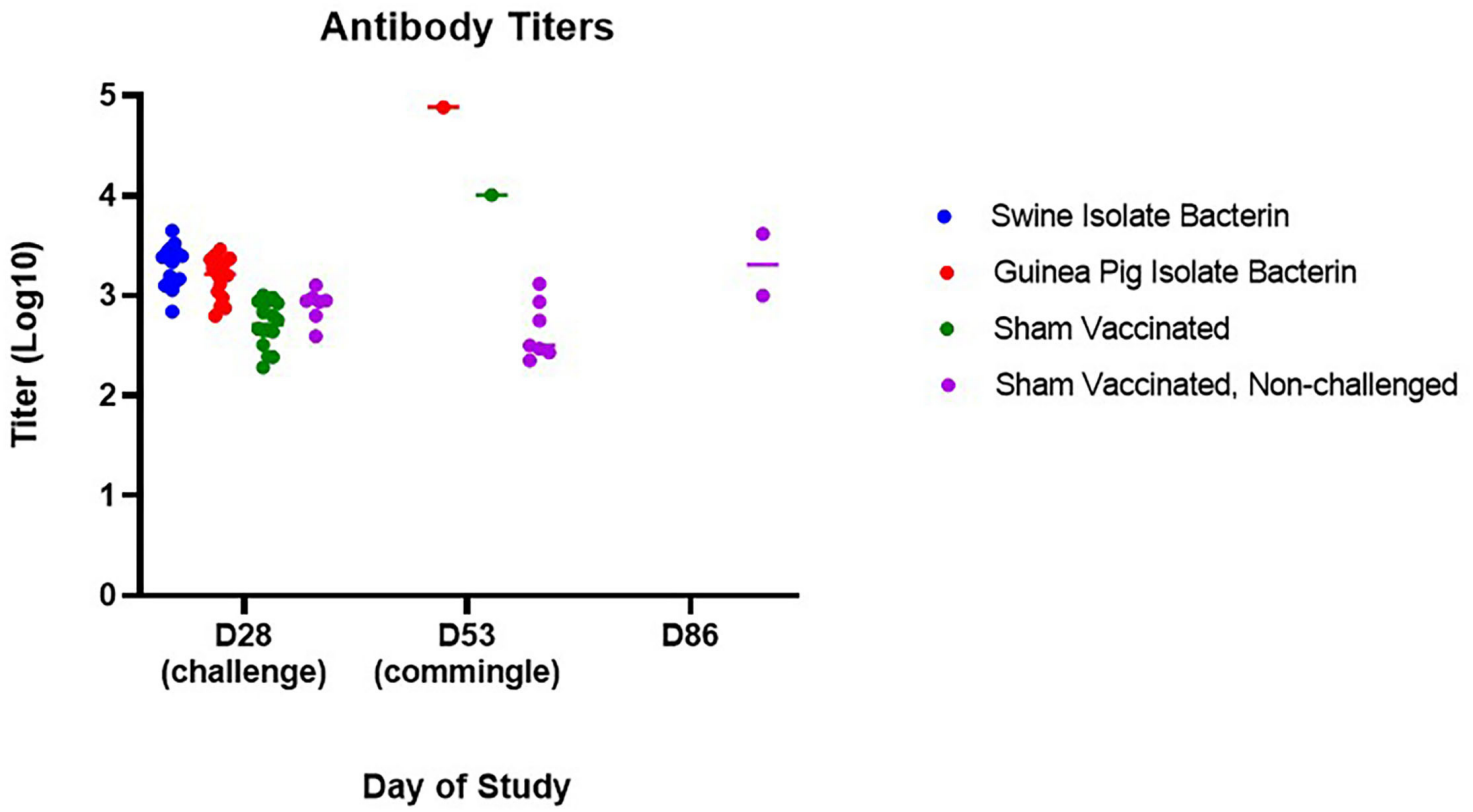
Serum antibody evaluation following challenge and commingling. Antibody titers for surviving animals were significantly higher than non-challenged animals 25 days post-challenge (day 53) (guinea pig isolate bacterin, *p* = 0.0009; sham vaccinated, *p* = 0.0110). Following commingling, an increase in titer was observed in surviving animals (*p* = 0.0177), although only one of the two surviving animals had a 4-fold increase in titer from D53 to D86.

To assess the risk surviving animals pose to naïve animals, three of the contact pigs were commingled with the surviving guinea pig bacterin vaccinated animal (group 2) and sham vaccinated animal (group 3) 25 days post-challenge (day 53). All contact pigs were febrile at one or more time points post-commingling (Figure 3). One pig had a mild fever (40.6°C) on day 4 post-commingling. Two contact pigs were febrile for multiple readings. One pig was febrile for 4 days beginning at 6 days post-commingling. The other contact pig became febrile 12 days post-commingling and developed clinical signs including depression, reluctance to rise, and cyanosis of extremities 15 days post-commingling. This pig was euthanized and SEZ was isolated from systemic samples. Evaluation of serum antibody titers 4 weeks post-commingling (day 86) revealed a 4-fold rise in titer for one of the two surviving contact animals following commingling (Figure 4), indicating exposure to SEZ.

## Discussion

SEZ causes severe disease in pigs when introduced into naïve herds. Pigs rapidly develop depression, anorexia, and high fever. Field mortality rates reach 30–50% (6). Experimental replication of disease revealed the susceptibility of healthy, conventionally raised pigs to SEZ which results in 100% mortality within 72 h of challenge (7). Because of the rapid and significant losses associated with SEZ introduction and the absence of approved vaccines to prevent infection, we investigated the use of autogenous bacterins to protect pigs against SEZ challenge.

Limited work has investigated preventing SEZ infection in pigs. The majority of this work has focused on subunit vaccines, used ATCC35246, a historic Chinese isolate, as the challenge strain, and evaluated protection in mice (8–12). Because of this, there are no data evaluating the serologic response to SEZ following bacterin vaccination, although the SEZ bacterin vaccine commercially available in China was used as a positive control in some studies (8–10). In this study, we observed a rise in titer following primary vaccination; however, there was no anamnestic response following boost vaccination. This is inconsistent with previous studies in our laboratory using whole cell bacterins adjuvanted with EmulsigenD, in which an anamnestic response to boost vaccination resulted in average titers > 10^4.7^ (13). The lack of anamnestic response following boost vaccination contributed to a lower average titer at challenge than has been seen previously with bacterin vaccination (swine isolate bacterin = 10^3.3^, guinea pig bacterin = 10^3.2^). Interestingly, although no anamnestic response was observed following boost vaccination, vaccinated pigs developed a febrile response the day after boost, indicating immune stimulation from the vaccine or adjuvant.

In contrast to previous reports showing success utilizing a commercially available SEZ bacterin vaccine in mice and pigs (8–10), the use of autogenous bacterin vaccines in this study did not provide protection against challenge with a 2019 SEZ isolate. This is likely associated with the comparatively low antibody titers induced by vaccination with the bacterins utilized in this study. Based on previous work in mice utilizing passive transfer of antibodies (8, 9, 11), there is strong evidence that the antibody response is important in protection against SEZ. The low titers induced by vaccination in this study may have been insufficient to provide protection and indicates titers over 10^3.5^ are required to prevent SEZ disease.

Multiple factors may be contributing to the reduced antibody response and lack of protection provided by the autogenous bacterins used in this study. One potential explanation is insufficient antigen included in the vaccine formulation. Though we utilized 10^9^ bacteria in each dose, which has been protective for other autogenous bacterins (13), it may be that SEZ requires a higher antigen load to stimulate a protective immune response. In addition, different strains were included in the vaccine formulation as compared with the commercial vaccine, which could impact vaccine efficacy. Another factor that may have influenced the antibody titers is the selected adjuvant. In this study, we utilized EmulsigenD, an oil-in-water adjuvant that has shown good efficacy previously when utilized in inactivated vaccines (13); however, adjuvant has been shown to play an important role in the development of the adaptive immune response (14, 15) and an alternative adjuvant may result in a different response. Previous work also utilized ATCC35246 as the challenge strain (8–12). While this isolate has a high genetic relatedness to the 2019 outbreak isolates (5), no comparison in virulence has been made and the 2019 isolates may have characteristics allowing them to better escape the adaptive immune response generated by vaccination. Finally, the use of swine as a SEZ model is uncommon and only one study utilized a SEZ bacterin to evaluate protection in pigs (10). That study utilized the SEZ vaccine commercially available in China and challenged intramuscularly with ATCC35246 at a challenge dose of 10^7^, which also could have contributed to the differences in observed outcomes between the studies.

In this study, we also evaluated surviving animals post-challenge for colonization and their potential to act as a reservoir for SEZ. We found surviving animals remained colonized over 3 weeks post-challenge and transmitted SEZ to naïve contact animals. This revealed pig-to-pig transmission of SEZ is possible from apparently healthy animals and provides evidence that heathy carrier animals may have been the source of SEZ at lairage leading to the high mortality events in 2019 (6). It also indicates that animals from herds stabilized following SEZ introduction can serve as a source of infection for naïve animals introduced into the herd. This emphasizes the importance of an effective control strategy for SEZ in continuous flow systems (4).

Currently, no vaccines are approved to prevent the severe disease or mortality associated with SEZ infection in pigs. Autogenous inactivated vaccines are commonly employed by the swine industry to combat endemic bacterial pathogens. The use of an autogenous bacterin containing 10^9^ bacteria and utilizing EmulsigenD as an adjuvant was not efficacious against challenge with a high mortality SEZ isolate from 2019. Continued work should investigate alternative vaccine formulations containing greater antigen loads or utilizing a different adjuvant, which could stimulate higher antibody titers and protect against SEZ challenge.

## Data Availability Statement

The original contributions presented in the study are included in the article/supplementary material, further inquiries can be directed to the corresponding author.

## Ethics Statement

The animal study was reviewed and approved by National Animal Disease Center Institutional Animal Care and Use Committee.

## Author Contributions

SH, AB, and SB contributed equally to study design, execution, and manuscript preparation. All authors contributed to the article and approved the submitted version.

## Conflict of Interest

The authors declare that the research was conducted in the absence of any commercial or financial relationships that could be construed as a potential conflict of interest.

## Publisher's Note

All claims expressed in this article are solely those of the authors and do not necessarily represent those of their affiliated organizations, or those of the publisher, the editors and the reviewers. Any product that may be evaluated in this article, or claim that may be made by its manufacturer, is not guaranteed or endorsed by the publisher.
